# A Trifecta of New Insights into Ovine Footrot for Infection Drivers, Immune Response, and Host-Pathogen Interactions

**DOI:** 10.1128/IAI.00270-21

**Published:** 2021-09-16

**Authors:** Adam M. Blanchard, Ceri E. Staley, Laurence Shaw, Sean R. Wattegedera, Christina-Marie Baumbach, Jule K. Michler, Catrin Rutland, Charlotte Back, Nerissa Newbold, Gary Entrican, Sabine Tötemeyer

**Affiliations:** a School of Veterinary Medicine and Science, University of Nottingham, Loughborough, Leicestershire, United Kingdom; b School of Science and Technology, Nottingham Trent University, Nottingham, United Kingdom; c Moredun Research Institute, Pentlands Science Park, Bush Loan, Penicuik, Midlothian, Scotland; d Institute of Anatomy, Histology and Embryology, Faculty of Veterinary Medicine, Leipzig University, Leipzig, Germany; Washington State University

**Keywords:** histology, host-pathogen interactions, metagenomics, metatranscriptomics, transcriptomics, veterinary microbiology

## Abstract

Footrot is a polymicrobial infectious disease in sheep causing severe lameness, leading to one of the industry’s largest welfare problems. The complex etiology of footrot makes *in situ* or *in vitro* investigations difficult. Computational methods offer a solution to understanding the bacteria involved and how they may interact with the host, ultimately providing a way to identify targets for future hypothesis-driven investigative work. Here, we present the first combined global analysis of bacterial community transcripts together with the host immune response in healthy and diseased ovine feet during a natural polymicrobial infection state using metatranscriptomics. The intratissue and surface bacterial populations and the most abundant bacterial transcriptomes were analyzed, demonstrating that footrot-affected skin has reduced diversity and increased abundances of not only the causative bacterium Dichelobacter nodosus but also other species such as Mycoplasma fermentans and Porphyromonas asaccharolytica. Host transcriptomics reveals the suppression of biological processes related to skin barrier function, vascular functions, and immunosurveillance in unhealthy interdigital skin, supported by histological findings that type I collagen (associated with scar tissue formation) is significantly increased in footrot-affected interdigital skin compared to outwardly healthy skin. Finally, we provide some interesting indications of host and pathogen interactions associated with virulence genes and the host spliceosome, which could lead to the identification of future therapeutic targets.

## INTRODUCTION

Ovine footrot is a persistent animal welfare issue and has a significant financial burden for farmers due to the costs of preventative footbaths, antibiotic treatments, and reduced carcass weights at slaughter (1). The causative bacterium Dichelobacter nodosus has received extensive attention since its description in the initiation of footrot (2). However, it has been accepted since the beginning of the 20th century that footrot is a polymicrobial disease, with Fusobacterium necrophorum, Spirochaeta penortha (3), Treponema podovis (4), and Corynebacterium pyogenes (5) being proposed as species that can exacerbate the lesions.

Currently, our understanding of bacterial populations associated with footrot is based only on 16S rRNA analysis from the skin surface (6, 7). The highly abundant genera identified in the footrot samples were congruent with those identified previously by standard microbiological techniques (*Corynebacterium*, *Fusobacterium*, *Dichelobacter*, and Treponema). However, additional genera were also identified (*Mycoplasma*, *Psychrobacter*, and *Porphyromonas*) (7), and their absence using traditional culture techniques could be due to the fastidious nature of the bacteria (8), or they were not yet identified (9). Investigating the total bacterial load within tissues, we have shown recently that in healthy tissues, bacterial loads are similar throughout the tissue depth and do not extend beyond the follicular depth in the reticular dermis. In contrast, in footrot samples, the bacterial load was highest in the superficial (or cornified) epidermal layers and decreased in the deeper layers but still beyond the follicular depth (10). This suggests that the infection allows further invasion by other species of bacteria to penetrate deeper into the interdigital tissue; however, these data were limited to the presence of bacteria based on universal primers not allowing species identification.

There is also a lack of information regarding host infection, how an immune response is mounted, and the species interactions. This area of investigation has recently benefited from the use of metatranscriptomics, a method of assessing host-pathogen interactions based on associated gene expression changes (11). The use of metatranscriptomics has been reviewed extensively (12); however, current published methods are based on, or optimizations of, cell culture models as developed in the original method article (11). The use of metatranscriptomics in natural polymicrobial infections is not as well reported. The first documented use was in relation to the onset of pediatric asthma (13) and oral disease (14, 15). However, its application to bovine digital dermatitis (BDD) (16), which has a clinical presentation and bacteria similar to those associated with footrot (17–20), highlighted its suitability to further our understanding of the intercellular microbial populations associated with agricultural diseases. Utilizing this experimental design, we have been able to determine the bacterial populations on the surface of the interdigital skin and within the deeper infected tissue, identify the differential expression of host transcripts, and elucidate interactions between the host and bacteria.

## RESULTS

### Sequence data.

**FIG 1:**
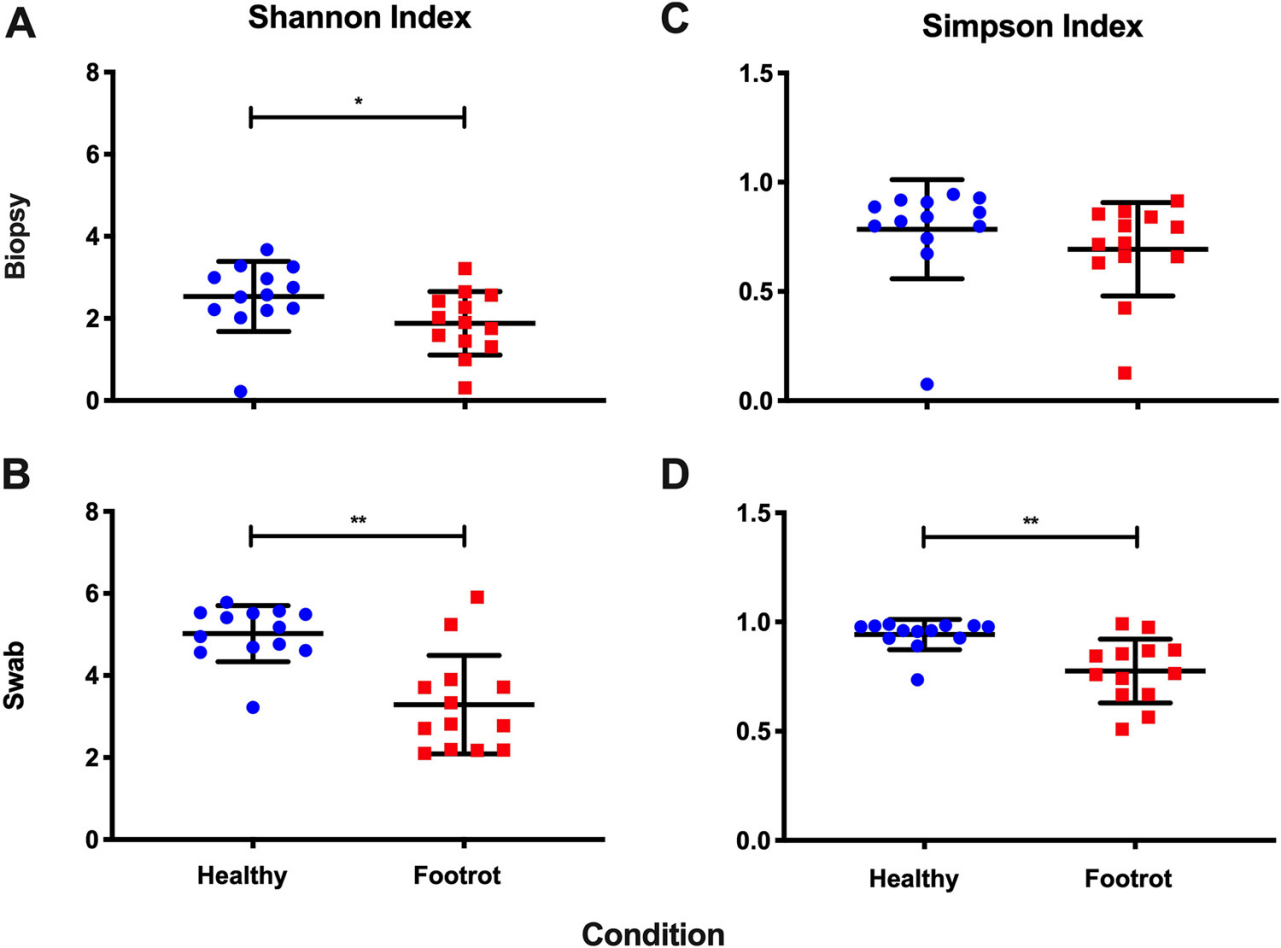
Diversity statistics for the biopsy and swab samples. (A) Shannon indices of biopsy samples; (B) Shannon indices of swab samples; (C) Simpson indices of biopsy samples; (D) Simpson indices of swab samples. Significant decreases were observed for footrot-affected samples for swabs using both Shannon and Simpson indices. A significant decrease in footrot-affected samples was observed only for biopsy specimens using the Shannon index. Statistical significance was calculated using a Mann-Whitney U test (*, *P* = 0.05; **, *P* = 0.005; ***, *P* = 0.0005).

Foot swabs and whole-thickness skin biopsy specimens were collected from sheep after slaughter that had at least one apparently healthy foot (*n* = 13) and one with signs of footrot (*n* = 13) to obtain matching samples from the same sheep. After quality filtering, there was an average of 8.7 million discordant ovine reads per sample to be used for bacterial taxonomic assignment from the foot biopsy specimens and 20.8 million discordant ovine reads for the accompanying swabs. All reads had an average Phred score of 40. To assess the overall diversity of the bacterial community from each sampling method, Shannon and Simpson indices were calculated. Using the Shannon index and calculating an equitability score (natural log of the species richness), representing the maximum expected diversity, revealed that healthy feet were highly diverse but that footrot feet showed a reduction in diversity (Table 1 and Fig. 1A). The Simpson indices also indicated that there was more diversity in the healthy samples, with an average value of 0.78, compared to 0.69 in the footrot biopsy specimens (Fig. 1B). This was furthermore reflected in the swabs, with an average value of 0.94 in the healthy samples, compared to 0.77 in footrot (Fig. 1). The Shannon index showed a significant difference between the two conditions for both biopsy specimens (*P* ≤ 0.005) and swabs (*P* ≤ 0.05), whereas only the swabs showed a significant difference for the Simpson index (*P* ≤ 0.005) (Fig. 1).

**TABLE 1:** Comparison of average calculated and maximum diversity values under each condition^*a*^

Condition	Biopsy specimens		Swab samples	
	Equitability value	Shannon index	Equitability value	Shannon index
Healthy	3.9	2.5	7.1	5.0
Footrot	3.3	1.9	7.1	3.2

a The data demonstrate the overall reduction in bacterial community diversity for footrot-affected individuals compared to the calculated maximum diversity expected from the data.

### Bacterial community.

**FIG 2:**
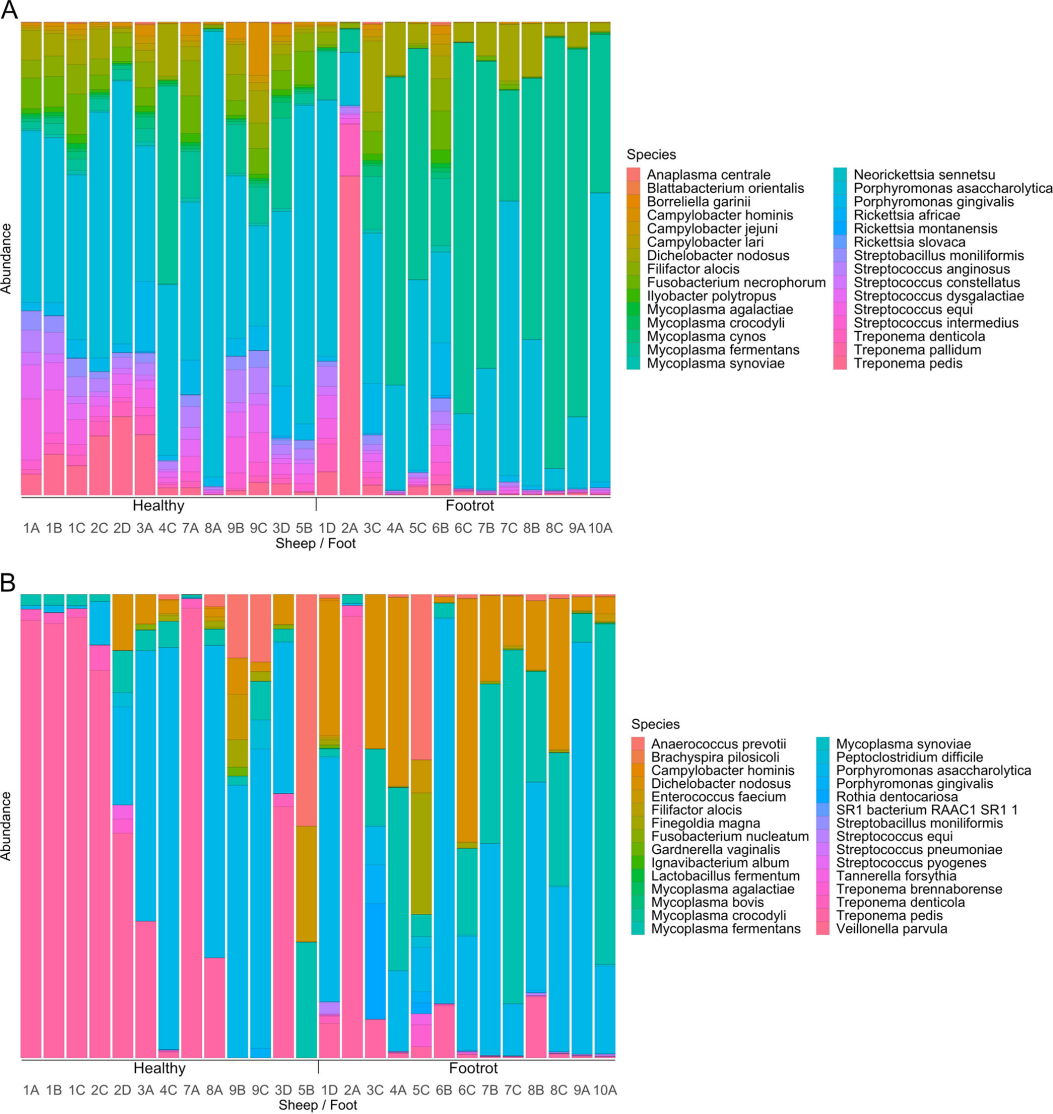
Species of bacteria identified as being increased in abundance in footrot-affected feet compared to healthy feet. (A) Top 30 species of bacteria in swab samples; (B) top 30 species of bacteria in biopsy samples.

Differences in abundance calculated between the two conditions were identified as samples having a >2-log fold change, with a false discovery rate (FDR) (Benjamini-Hochberg [21])-corrected *P* value of <0.05 and where average counts had a difference of >10 (full taxonomic assignments are available in Table S1 in the supplemental material for swabs and Table S2 for biopsy specimens). In swabs, 20 species of bacteria were found in significantly increased abundances in footrot samples (Fig. 2). These included Treponema pedis, Treponema denticola, D. nodosus, and F. necrophorum, all known to cause various foot diseases in sheep. Among the bacterial species found in significantly reduced abundances in footrot samples were 10 species of Staphylococcus spp., Bacillus licheniformis, Parageobacillus thermoglucosidasius, and Nocardiopsis alba. All differential abundance data for the swabs are available in Table S3.

Applying the same criteria to biopsy specimens, three species of bacteria were found in differential abundances between the two conditions, namely, D. nodosus, Mycoplasma fermentans, and Porphyromonas asaccharolytica. D. nodosus had the greatest and most significant increase in footrot biopsy specimens, with a log fold change increase of 7.0 (*P* = 1.89E−06); additionally, M. fermentans had a log fold increase of 6.2 (*P* = 2.59E−05) and P. asaccharolytica had a log fold increase of 3.5 (*P* = 0.018) in footrot biopsy samples. No species were found to be significantly decreased between the two conditions in the biopsy specimens (all differential abundance data for the biopsy specimens are available in Table S4). Although some archaea were identified in both biopsy and swab samples, none were significantly more or less abundant in footrot-affected feet than in healthy feet.

As short-read sequencing has limitations in identifying bacteria to the species level, the bacteria most significantly increased in abundance in footrot-affected tissues were confirmed to be D. nodosus, M. fermentans, and *P. asaccharolytica* by specific quantitative PCR (qPCR), species-specific PCR, and PCR followed by sequencing, respectively. In addition, F. nucleatum was identified as the only *Fusobacterium* species; however, qPCR demonstrated that this was a misidentification, and F. necrophorum was present as expected.

### Comparative analysis of tissue and surface bacterial communities.

Comparison of the bacterial community compositions between biopsy specimens and swabs was carried out to assess if swabs act as a good, noninvasive method to assess bacterial presence or absence. The taxonomic assignments from both swab and biopsy specimen data were tested to ascertain whether a clear relationship existed between taxonomic assignments for the same sheep using both the correlation and similarity hypothesis tests outlined in Materials and Methods. Under the null hypothesis for the correlation test, there was no correlation between swab and tissue samples. A *P* value of 0.0301 was obtained, providing strong evidence of a relationship. However, it should be noted that this is evidence of a relationship in the presence of bacterial species between biopsy and swab samples rather than them containing the same species.

To test the latter claim, the similarity test outlined in Materials and Methods was used. Here, the null hypothesis was that biopsy and swab samples reveal the presence of the same bacteria. This test produced a conservative *P* value of <10^−5^, providing overwhelming evidence that swab and biopsy samples from the same sheep do not contain the same species of bacteria. Specifically, two random biopsy samples will have more species in common than a swab and a biopsy sample from the same sheep.

### Differential expression of proinflammatory mediators in healthy versus footrot-affected interdigital skin.

Based on the calculated β-value and associated adjusted *P* value from the differential expression analysis, the transcripts that showed increased expression in footrot-affected interdigital skin were a large number of proteins important for barrier function. These included proteins involved in collagen production and collagen binding (procollagen C-endopeptidase enhancer 2 [PCOLCE2], collagen type VI alpha 6 chain, collagen type XXIII alpha 1 chain, and keratocan/lumican [collagen-binding leucine-rich proteoglycans widely distributed in interstitial connective tissues]), cell-cell adhesion (cadherin 3 [CDH3], CDH19, and pro-CDH10 [PCDH10]), maintenance of cell junctions (GJB4), and long-chain fatty acid synthesis (fatty acid elongase 7 [ELOVL7], ELOVL3, acyl-CoA synthetase bubblegum family member 1 [ACSBG1], and acyl-CoA wax alcohol acyltransferase 1 [AWAT1]). In addition, transcripts involved in immunosurveillance, such as the scavenger receptors SCARA5 and SSC5D, were more highly expressed in footrot-affected samples (see Table 2 for the top 25 transcripts and Table S5 for all transcripts). In contrast, transcripts that showed lower expression levels than those in healthy interdigital skin include cytokines involved in wound healing (interleukin-19 [IL-19] and IL-20) and keratinocyte proliferation/differentiation (IL-6 and leukemia inhibitory factor [LIF]); epithelial cell-derived chemokines that recruit monocytes (CCL2), lymphocytes (CCL20), and neutrophils (CXCL1 and CXCL8); and prostaglandin-endoperoxide synthase 2 (PGE2/COX2), which is also involved in skin wound healing. Another group of significantly decreased transcripts includes matrix metalloproteases (MMP1, MMP3, MMP9, MMP13, MMP20, tenascin C [TNC], and tissue inhibitor of metalloprotease 1 [TIMP1]) and their regulators (SERPINE1, ADAMTS4, and ADAMTS16) associated with chronic wounds and collagen turnover (see Table 3 for the top 25 transcripts and Table S5 for all transcripts).

**TABLE 2:** Top 25 differentially more highly expressed genes in footrot-affected skin than in healthy skin^*a*^

Gene	Description	β-Value	SE	*q* value
ELOVL7	ELOVL fatty acid elongase 7	2.812237	0.884427	0.035516
MNT	MAX network transcriptional repressor	2.415885	0.773256	0.038156
FRAS1	Fraser extracellular matrix complex subunit 1	2.358302	0.738887	0.034774
CA6	Carbonic anhydrase 6	2.273248	0.686033	0.029014
ATP13A4	ATPase 13A4	2.261712	0.648362	0.023149
PNPLA5	Patatin-like phospholipase domain-containing 5	2.230351	0.627058	0.021215
ELOVL3	ELOVL fatty acid elongase 3	2.070317	0.598268	0.02425
STMN2	Stathmin 2	2.021979	0.464736	0.007856
NOS1	Nitric oxide synthase 1	2.004654	0.546986	0.018297
ACSBG1	Acyl-CoA synthetase bubblegum family member 1	1.933404	0.590342	0.030742
AWAT1	Acyl-CoA wax alcohol acyltransferase 1	1.849357	0.543633	0.02594
CCL26	C-C motif chemokine ligand 26	1.842727	0.621591	0.046882
CCDC155	Coiled-coil domain-containing 155	1.791928	0.584831	0.041351
PI16	Peptidase inhibitor 16	1.765247	0.400402	0.007477
FAR2	Fatty acyl-CoA reductase 2	1.711846	0.519782	0.02993
PTX4	Pentraxin 4	1.681009	0.336588	0.004979
LRRC36	Leucine-rich repeat-containing 36	1.669794	0.465845	0.020504
AGTR1	Angiotensin II receptor type 1	1.661825	0.442673	0.016379
GALNT8	Polypeptide *N*-acetylgalactosaminyltransferase 8	1.631845	0.457915	0.020974
CYP2F1	Cytochrome P450 family 2 subfamily F member 1	1.55567	0.44436	0.022765
TOGARAM2	TOG array regulator of axonemal microtubules 2	1.530427	0.48021	0.035066
DNASE1L2	Deoxyribonuclease 1-like 2	1.515543	0.406144	0.0169
FAM221A	Family with sequence similarity 221 member A	1.506933	0.378682	0.01257
AQP9	Aquaporin 9	1.506146	0.384521	0.013256
DGAT2L6	Diacylglycerol *O*-acyltransferase 2-like 6	1.505104	0.480771	0.03797

a *q* value, Benjamini-Hochberg FDR-adjusted *P* value; β-value, bias estimator analogous to a fold change.

**TABLE 3:** Top 25 more lowly expressed genes in footrot-affected interdigital skin than in healthy skin^*a*^

Gene	Description	β-Value	SE	*q* value
IL-19	Interleukin-19	−2.96775	0.804409	0.017781
PTGS2	Prostaglandin-endoperoxide synthase 2 (PGE2/COX2)	−2.8683	0.75813	0.015736
MMP3	Matrix metalloprotease 3	−2.71059	0.616087	0.007597
IL-6	Interleukin-6 precursor	−2.4267	0.568937	0.008635
IL-20	Interleukin-20	−2.30205	0.758643	0.04305
A2ML1	Alpha-2-macroglobulin-like 1	−2.23879	0.765254	0.049443
CCL20	C-C motif chemokine ligand 20	−2.18479	0.501858	0.007856
CXCL8	Interleukin-8	−2.10659	0.685411	0.040981
SLPI	Antileukoproteinase precursor	−2.04964	0.672506	0.042275
MGAM	Maltase-glucoamylase	−2.02022	0.568845	0.021334
MARCKSL1	MARCKS-like 1	−1.95955	0.631205	0.039119
MEFV	Pyrin innate immunity regulator	−1.90552	0.514121	0.017474
MMP13^*b*^	Matrix metalloprotease 13	−1.83676	0.523263	0.022523
ADAMTS16	ADAM metallopeptidase with thrombospondin type 1 motif 16	−1.80019	0.479709	0.016379
ACOD1	Aconitate decarboxylase 1	−1.68652	0.563289	0.045291
PTX3	Pentraxin 3	−1.66311	0.446328	0.016904
MMP13^*b*^	Matrix metalloprotease 13	−1.65481	0.561768	0.048097
ADAMTS4	ADAM metallopeptidase with thrombospondin type 1 motif 4	−1.59355	0.394111	0.011418
MMP1	Matrix metalloprotease 1	−1.55518	0.37913	0.01037
FOSL1	FOS-like 1, AP-1 transcription factor subunit	−1.51685	0.383897	0.012932
MGAT3	Mannosyl (β-1,4-)-glycoprotein β-1,4-*N*-acetylglucosaminyltransferase	−1.46074	0.377507	0.014131
CCL2	C-C motif chemokine ligand 2	−1.40425	0.325691	0.008232
CSF3	Colony-stimulating factor 3	−1.4015	0.473053	0.047061
PLAUR	Urokinase plasminogen activator surface receptor precursor	−1.36363	0.322566	0.009128

a *q* value, Benjamini-Hochberg FDR-adjusted *P* value; β-value, bias estimator analogous to a fold change.

b Splice variant.

### Biological process enrichment.

Using Block-correlated coupled cluster (BCCC) biclustering and associated Gene Ontology (GO) biological processes, the genes and conditions were grouped into a total of 32 clusters. There were 2,531 genes over 20 samples that clustered showing upregulated biological processes, including significant positive regulation. The top 15 were positive regulation of transcription (*n* = 106; *P* = 2.94E−26), protein folding (*n* = 34; *P* = 3.5E−21), regulation of DNA-templated transcription (*n* = 121; *P* = 4.51E−21), metabolic processes (*n* = 61; *P* = 1.44E−18), DNA-templated transcription (*n* = 63; *P* = 1.48E−18), rRNA processing (*n* = 20; *P* = 9.12E−15), protein transport (*n* = 32; *P* = 2.08E−13), ribosome biogenesis (*n* = 17; *P* = 2.20E−13), osteoblast differentiation (*n* = 24; *P* = 4.01E−12), transcription from the RNA polymerase II promoter (*n* = 49; *P* = 5.84E−12), negative regulation of transcripts from the RNA polymerase II promoter (*n* = 63; *P* = 7.75E−12), negative regulation of apoptotic processes (*n* = 46; *P* = 1.91E−11), positive regulation of telomerase RNA localization to the Cajal body (*n* = 10; *P* = 9.34E−11), proteolysis (*n* = 63; *P* = 1.65E−10), and translational initiation (*n* = 16; *P* = 1.74E−10).

There were 541 genes over 17 samples that clustered showing significant downregulation of biological processes in footrot-affected samples. Cluster 1 showed decreases in epidermis development (*n* = 17; *P* = 2.3E−06), multicellular organismal water homeostasis (*n* = 8; *P* = 1.8E−05), peptidoglycan catabolic processes (*n* = 4; *P* = 4.6E−05), antimicrobial humoral responses (*n* = 7; *P* = 9.6E−05), tissue development (*n* = 41; *P* = 1.1E−04), monovalent inorganic cation homeostasis (*n* = 8; *P* = 8.2E−4), defense responses to bacteria (*n* = 11; *P* = 8.2E−04), fatty acid metabolic processes (*n* = 12; *P* = 1.3E−03), polyol transport (*n* = 3; *P* = 2.7E−03), skin development (*n* = 11; *P* = 2.7E−03), water transport (*n* = 4; *P* = 3.4E−03), and regulation of pH (*n* = 6; *P* = 3.4E−03). Cluster 2 showed downregulation for neutrophil chemotaxis (*n* = 7; *P* = 4.3E−06), myeloid leukocyte migration (*n* = 9; *P* = 7.2E−06), leukocyte migration (*n* = 11; *P* = 9.8E−06), cell chemotaxis (*n* = 10; *P* = 2.6E−05), defense responses (*n* = 19; *P* = 2.8E−05), immune system processes (*n* = 26; *P* = 4.3E−05), chemotaxis (*n* = 12; *P* = 1.2E−04), antimicrobial humoral responses (*n* = 5; *P* = 1.2E−04), immune responses (*n* = 18; *P* = 1.2E−04), and responses to an external stimulus (*n* = 23; *P* = 1.5E−04). The third cluster showed downregulation for *S*-adenosylhomocysteine catabolic processes (*n* = 2; *P* = 5.7E−04) alone.

Investigating KEGG pathway enrichment also identified cytokine-cytokine receptor interactions (*n* = 26; *P* = 4.5E−6), the IL-17 signaling pathway (*n* = 13; *P* = 7.7E−08), and the tumor necrosis factor (TNF) signaling pathway (*n* = 12; *P* = 7.7E−05) to be downregulated in the footrot-affected samples, while steroid hormone biosynthesis (*n* = 7; *P* = 2.4E−04) and ribosome biogenesis (*n* = 31; *P* = 4.07E−08) were upregulated.

### Putative host-pathogen interactions.

To assess the expression of bacterial genes, the bacterial RNA reads were aligned against the reference bacterial transcriptomes that were identified as those traditionally associated with ovine foot disease (D. nodosus, F. necrophorum, T. pedis, and T. denticola) and those additionally found to be the most differentially abundant in the footrot biopsy samples (M. fermentans and *P. asaccharolytica*). These data were then used, along with the sheep expression data, to understand correlations and host and putative pathogen interactions (Table S6).

The interactions were calculated with an FDR-adjusted *P* value using the Benjamini-Hochberg procedure. Due to the stringency of this multiple-test adjustment, no significance was determined. However, the raw *P* values were low (in some cases <0.00005); therefore, these data were investigated further but only as an indicative positive bacterium/gene correlation. Based on a raw *P* value of <0.00005, there were four sheep transcripts that were associated with five D. nodosus genes (Table 4). From D. nodosus, aminoacyl-histidine dipeptidase, acidic extracellular subtilisin-like protease precursor (AprV5), outer membrane protein 1E, bacterial extracellular solute-binding protein, and aminoacyl-histidine dipeptidase were identified to correlate with small nucleolar RNA, the C/D box, U6 spliceosomal RNA, synapsin, and U6 spliceosomal RNA from Ovis aries. There were more correlations between M. fermentans and Ovis aries, with a total of 15 bacterial transcripts associated with four host transcripts, where the raw *P* value was 0.0005. The bacterial transcripts were shown to be overwhelmingly responsible for cellular transport on both the host and pathogen sides. There were a further three bacterial and sheep interactions in *T. pedis* (*P* = 0.00005), which suggested that the bacterial flagellin and host membrane protein and a bacterial hypothetical protein and putative lipoprotein correlate with a sheep microRNA (miRNA) (Table 4).

**TABLE 4:** Correlations between bacterial and host gene expression

Bacterial species	NCBI protein accession no.	Function	Ovis aries transcript accession no.	Function^*a*^	*P* value
D. nodosus	ABQ13122.1	Aminoacyl-histidine dipeptidase	ENSOART00000026163	Small nucleolar RNA, C/D box	1.66E−05

D. nodosus	ABQ13667.1	Acidic extracellular subtilisin-like protease precursor (AprV5)	ENSOART00000023789	U6 spliceosomal RNA	7.10E−05

D. nodosus	ABQ13351.1	Outer membrane protein 1E	ENSOART00000014155	Synapsin	7.49E−05
D. nodosus	ABQ13881.1	Bacterial extracellular solute-binding protein			7.49E−05

D. nodosus	ABQ13122.1	Aminoacyl-histidine dipeptidase	ENSOART00000023789	U6 spliceosomal RNA	7.49E−05

M. fermentans	WP_013526775.1	Protein translocase subunit	ENSOART00000015973	Proplatelet basic protein	2.88E−05

M. fermentans	WP_013526734.1	Putative oligopeptide ABC transporter, ATP-binding protein	ENSOART00000022767	Novel transcript	4.71E−05

M. fermentans	WP_013526734.1	DNA-directed RNA polymerase subunit beta	ENSOART00000013696	Potassium voltage-gated channel	7.04E−05

M. fermentans	WP_013526778.1	ATP synthase subunit beta	ENSOART00000017736	Novel transcript	7.04E−05

M. fermentans	ADV34079.1	MgpA-like protein	ENSOART00000013889	CXXC-type zinc finger 1 CPG-binding PHD finger	9.28E−05
M. fermentans	WP_013354336.1	Bifunctional oligoribonuclease/PAP phosphatase			9.28E−05
M. fermentans	WP_013526633.1	Adenine phosphoribosyltransferase			9.28E−05
M. fermentans	ADV34286.1	Oligopeptide ABC transporter permease protein			9.28E−05
M. fermentans	WP_013354483.1	ABC transporter permease			9.28E−05
M. fermentans	WP_013354556.1	Sugar ABC transporter permease			9.28E−05
M. fermentans	ADV34629.1	NADPH flavin oxidoreductase			9.28E−05
M. fermentans	WP_013354747.1	Nitroreductase family protein			9.28E−05
M. fermentans	ADV34954.1	Transcription antitermination protein			9.28E−05
M. fermentans	WP_013527166.1	ABC transporter ATP-binding protein			9.28E−05
M. fermentans	WP_013527168.1	ABC transporter permease			2.88E−05

*T. pedis*	WP_024465740.1	Flagellin	ENSOART00000022912	Novel membrane protein	5.38E−05

*T. pedis*	WP_051150643.1	Hypothetical protein	ENSOART00000026139	ncRNA (miRNA)	5.47E−05
*T. pedis*	AGT42887.1	Putative lipoprotein			5.47E−05

a ncRNA, noncoding RNA.

The correlations between the sheep transcripts and the bacterial transcripts from F. necrophorum, *P. asaccharolytica*, and T. denticola had raw *P* values of <0.0005, <0.001, and <0.004, respectively. Although low, the *P* values with the number of tests being performed were not investigated any further (full data are available in Table S6).

### Collagen composition differs in the dermis of healthy and footrot tissues.

**FIG 3:**
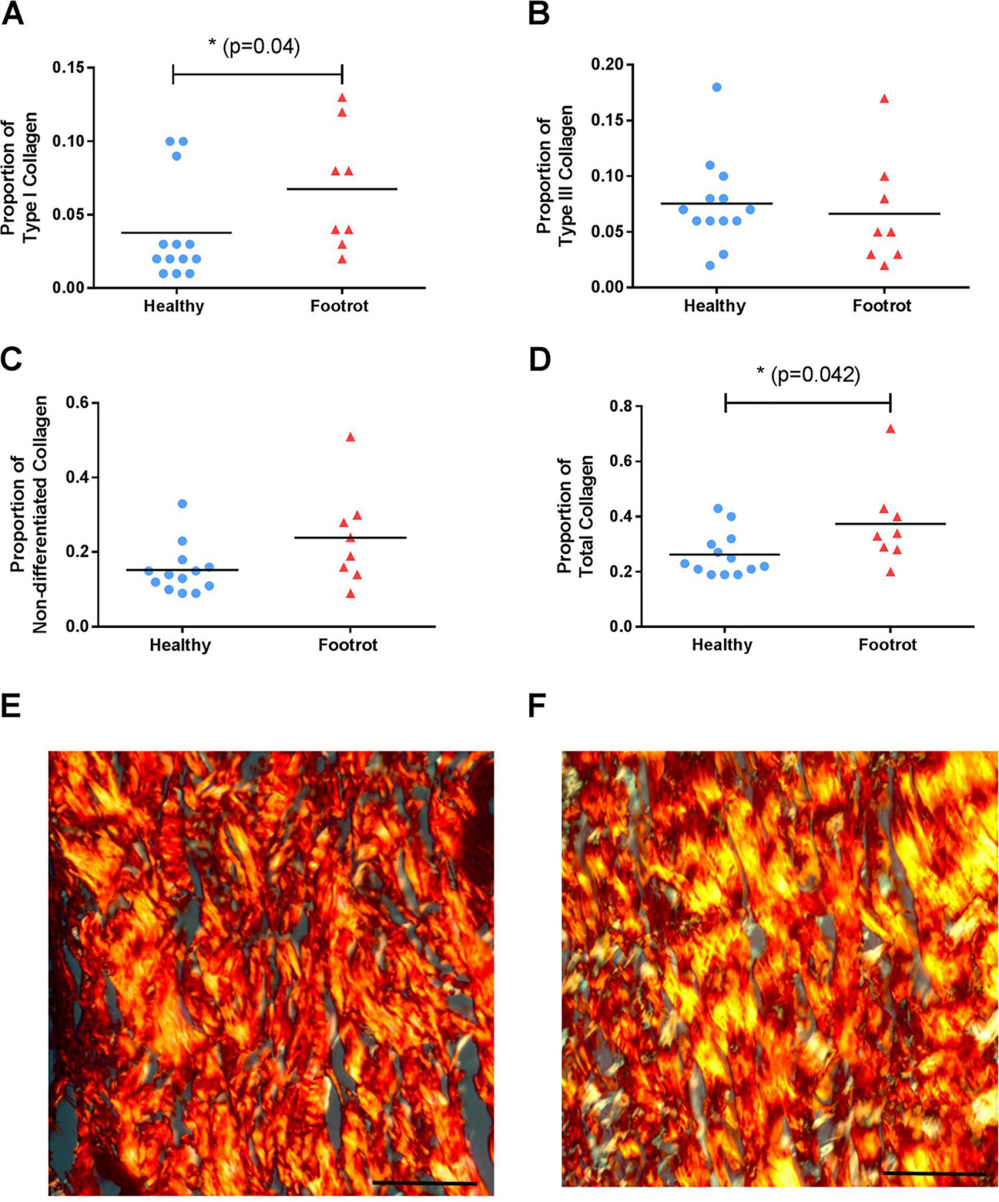
Collagen expression in healthy and footrot ovine interdigital skin dermis. Picrosirius histological staining was used to differentiate and quantify collagens in healthy (*n* = 13) and footrot (*n* = 8) samples. (A to D) Proportions of type I collagen (A), type III collagen (B), undifferentiated collagens (C), and total collagen (D). (E and F) Representative photomicrographs showing picrosirius staining under phase microscopy from healthy (E) and footrot (F) samples. Type I collagen stained yellow, type III collagen stained green, and undifferentiated collagen stained red. Bars, 50 μm. Significance is designated by an asterisk on a straight line, with the *t* test result defined as a *P* value of *≤*0.05.

Since the collagen composition changes in scar tissue formation, picrosirius (PS)-stained tissue sections were used to differentiate collagen types I and III from each other and other collagen types. To investigate whether there were any differences in the collagen compositions of the dermis, the proportions of type I, type III, nondifferentiated, and total collagen were calculated (Fig. 3E and F). The proportions of total and type I collagen were significantly increased in the dermis of footrot samples compared to healthy samples (*P* = 0.04 and 0.042, respectively) (Fig. 3A and D). The proportions of nondifferentiated and type III collagen were not significantly different in the dermis layers of healthy and footrot-affected tissues (Fig. 3B and C).

## DISCUSSION

D. nodosus was established as the causative bacterium of ovine footrot in the 1940s, and it has long been accepted that F. necrophorum plays a role in the disease etiology (4). However, in our work, we have identified additional common core species that are also associated with footrot lesions (M. fermentans and *P. asaccharolytica*). Furthermore, we have shown putative interactions with the molecular host defense systems during infection in bacterial species identified as highly abundant in footrot and even those without a significant difference in abundance between healthy and footrot-affected tissues, such as *T. pedis*. Using paired biopsy specimens collected from footrot-affected sheep at the point of slaughter, we were able to comprehensively show that skin swabs are a poor proxy for identifying which bacteria are present in the tissue. This is potentially due to bacterial contamination present from environmental sources such as feces and soil collected during transport and grazing. However, we have shown that biopsy specimens provide an intradermal approach to reproducibly assessing differences between individual animals in an invasive infection like footrot. The biopsy approach also enables the ability to differentiate between live and dead bacterial cells, showing species that could be playing an active role in the disease rather than nonviable environmental contamination, which could dominate results gathered from skin surface swabs.

### The bacterial community structure of footrot.

There are inherent limitations to taxonomic assignments of bacteria to the species level based on sequence read alignments; however, metagenomics allows the most accurate and informative assessment of environmental niches. Metatranscriptomics also allows the analysis of viable and live bacteria due to the rapid degradation of RNA in dead cells. Previous studies have shown the ovine interdigital bacterial community structure using 16S rRNA genes, with predominant bacterial genera identified as *Mycoplasma* spp., *Corynebacterium* spp., *Psychrobacter* spp., Treponema spp., Staphylococcus spp., *Peptostreptococcus* spp., and *Dichelobacter* spp. (6, 7). The results from this study are highly congruent with what has previously been identified; however, due to the higher taxonomic sensitivity afforded by metagenomics and metatranscriptomics, we have been able to classify these bacterial genera to the species level. Those with differential abundances associated with footrot found on the skin surface were identified as *T. pedis*, T. denticola, D. nodosus, and F. necrophorum. These most abundant species were confirmed by PCR or qPCR to further increase the validity of the sequence data assignments. These species are commonly found with other ovine foot diseases, such as contagious ovine digital dermatitis (CODD) (22) and interdigital dermatitis (ID) (7), and the bovine foot disease bovine digital dermatitis (BDD) (18). The species with differential abundances associated with footrot intradermally were D. nodosus, M. fermentans, and *P. asaccharolytica*. Given that D. nodosus is a poor pathogen and often requires tissue damage and the presence of other bacteria for infection, it stands to assume that *P. asaccharolytica* and M. fermentans may also have an important role in disease susceptibility. These differences between healthy and footrot-affected feet also extended beyond the presence and absence of species to the overall bacterial diversity, with a significant drop in footrot samples. This reduction in diversity has been mirrored in CODD (22).

### Host response and pathogen interactions.

Investigation of the host-pathogen interactions through correlation analysis has identified some interesting associations that warrant further investigations. The most promising appears to be the association between the virulence gene *aprV5* and the Ovis aries transcript U6 spliceosomal RNA. This particular noncoding small nuclear RNA (snRNA) is responsible for catalyzing the excision of introns and is a major aspect of posttranslational modifications, with the ability to alter the structure, function, and stability of the translated protein. In the case of infections, some species of bacteria have been implicated in hijacking the host splicing machinery and altering the splicing pattern, leading to the perturbation of the host response (23, 24). Despite the lack of knowledge about the mechanism, there is evidence that certain *Listeria*, Salmonella, and Mycobacterium species have the ability to produce factors that have a direct or indirect impact on the regulation of alternative splicing (24–26). Alternative splicing from the U6 spliceosomal RNA can interfere with the normal activation of T cells and B lymphocytes and the regulation of the signaling in several Toll-like receptors (TLRs) (TLR2, TLR3, and TLR4) (27), which could tie in with certain pathways (monovalent inorganic cation homeostasis, the defense response to bacteria, skin development, neutrophil chemotaxis, leukocyte migration, the defense response, immune system processes, and the immune response) that were identified as being downregulated in these data.

The acidic extracellular protease gene *aprV5* is associated with the correct cleavage of the other proteases secreted by D. nodosus, AprV2 and BprV, to their mature active form (28). Whereas the closely related AprV2 acidic protease is a known virulence factor responsible for elastase activity and its degradation of the host extracellular matrix (29), the role of AprV5 in footrot is unclear. The abundance of D. nodosus isolates with *aprV5* has been shown to be around 25 % from clinically affected farms and lacks any clear delineation toward disease severity (30). However, as other bacteria possess other mechanisms of action on snRNPs, it may be an interesting focus of future studies.

### Sheep interdigital skin microbiota and scar tissue formation in footrot.

The host response to the skin microbiota has to be carefully regulated, as innocuous microbes and host surveillance at epithelial barriers are in constant proximity. In healthy tissues, bacteria are located predominantly in the epidermis, while tissue damage and invasive bacteria such as D. nodosus allow access of bacteria to deeper dermal tissue layers (10). The ovine host response to footrot demonstrated through the differential expression of a range of transcripts involved in proinflammatory mediation (the cytokines IL-19, IL-20, IL-6, and LIF and the chemokines CCL2, CCL20, CXCL1, CXCL8, and prostaglandin-endoperoxide synthase 2 [PGE2/COX2]) and of matrix metalloproteases (MMP1, MMP3, MMP9, MMP13, TNC, and TIMP1) and, interestingly, their regulators (SERPINE1, ADAMTS4, and ADAMTS16) are all associated with wound healing, collagen turnover, and scar tissue formation. Collagen I was detected significantly more frequently in diseased dermis than in noninfected dermal tissue, leading to the conclusion that infection or coinfection clearly induces current, or ongoing, scar formation in the dermis.

The process of secondary-intention wound healing with scar formation is classically divided into three main overlapping phases: inflammation, proliferation, and remodeling. The cytokines and chemokines IL-6, CCL2, CXCL1, and CXCL8, identified as being differentially expressed in response to footrot, are associated with acute inflammation in response to tissue injury (31, 32). The IL-20 cytokine family (IL-19, IL-20, IL-22, IL-24, and IL-26) contributes to various stages of this wound-healing process: they are primarily secreted by infiltrating innate immune cells and lymphocytes shortly after an injury and preferentially stimulate keratinocytes to secrete antimicrobial peptides, chemokines, and vascular endothelial growth factor A (VEGFA), which in turn promotes angiogenesis (33). Surprisingly, we observed significantly reduced expression of IL-19 and IL-20 transcripts in footrot samples. This was accompanied by the reduced expression of secretory leukocyte protease inhibitor 1 (SLP1), a protein essential for optimal wound healing due to its antimicrobial and anti-inflammatory properties (34). Matrix metalloproteases (MMPs) are crucial for extracellular matrix degradation and deposition, which is essential for wound reepithelialization and during tissue remodeling. MMP expression and activity are tightly controlled during wound healing, at the expression level and through endogenous tissue inhibitors of metalloproteases (TIMPs); specific MMPs are confined to particular locations in the wound and to specific stages of wound repair (35).

The dysregulation of MMPs leads to prolonged inflammation and delayed wound healing (36). In footrot-affected tissues, we observed differential expression of the collagenases MMP1 and −13, the gelatinase MMP9, and the stromalysin MMP3. We observed reduced expression compared to that in uninfected interdigital skin tissue, which would impact the ability of infected interdigital skin tissue to heal. In contrast, the increased expression of factors essential for skin barrier function, such as fatty acid elongases (ELOVL7, ELOVL3, and ACSBG1), and of proteins involved in collagen production and collagen binding in response to footrot suggests that some level of skin regeneration is ongoing (36, 37).

### Putative role of bacterial communities in footrot.

One of the bacteria significantly increased in abundance on footrot-infected lesions, M. fermentans, might affect the ability of host skin cells to respond to bacterial infection. M. fermentans is usually considered a human commensal or opportunistic pathogen (38); however, it has been isolated from genital ulcers in sheep (39), with the possibility of urine and fecal contamination causing transmission to the infected hooves. Chronic infections of monocytes and macrophages with intracellular low-pathogenicity *Mycoplasma* spp. have been shown to impair their inflammatory response to live bacteria and bacterial products (40, 41). That we see higher MMP transcript levels in outwardly healthy interdigital skin is in contrast to the lack of detection of MMP RNA in healthy human or murine skin (42). However, this is consistent with the marked expression of the inflammatory cytokines/chemokines IL-1β, IL-6, and CXCL8 in outwardly healthy ovine interdigital skin, which might be due to the constant environmental changes and pressures impacting interdigital skin or might be associated with subclinical disease that may have developed into ID and footrot in the future (7). During the remodeling phase of scar tissue formation, initially deposited collagen III molecules are gradually replaced by type I collagen, and their orientation becomes more organized (37). Mature cutaneous scars consist of 80 to 90 % type I collagen arranged in parallel bundles (43). This particular orientation as well as less pronounced or missing rete ridges weaken the strength of the scar tissue compared to normal skin in humans to only 70 to 80 % (37). This renders the tissue more susceptible to injury and trauma, which are suspected predisposing factors of footrot. The latter might also contribute to the frequently observed relapses and underlines not only the polymicrobial but also the multifactorial etiology of footrot. For BDD, a dysfunctional skin barrier and disturbed tissue integrity are even hypothesized to be essential prerequisites for infection altogether since experimental disease models without skin maceration prior to infection fail to mirror naturally occurring BDD lesions appropriately (44).

Currently, there is conflicting evidence of the impact of the microbiome on wound healing, with some evidence of host commensal interactions promoting wound healing, while colonization of pathogenic bacteria may invade deeper into tissues or lead to chronic infections and biofilm formation (45, 46). In the context of footrot, we identified another bacterial species in addition to D. nodosus that is associated with footrot and also known to be a synergistic wound pathogen, *P. asaccharolytica*. When present in combination with anaerobic and aerobic bacteria such as Prevotella melaninogenica, Peptostreptococcus micros, and Klebsiella pneumoniae, *P. asaccharolytica* exacerbates the disease process (47–49). While antibiotic injections will affect indiscriminately commensal and pathogenic bacteria, the effectiveness of parenteral antibiotics in footrot demonstrates their high impact on pathogenic bacteria, leading to swift recovery in most cases (50). Interestingly, resistance genes against tetracycline, the most commonly used antibiotic against footrot, have so far not been identified in D. nodosus genome sequences, suggesting that the antibiotic treatment mainly affects other microbes of that polymicrobial infection, finally enabling the host immune system to eliminate D. nodosus.

### Conclusion.

Ovine footrot is a complex polymicrobial disease, and there is a clear need to further elucidate the intricate host-microbe interactions. We aimed to investigate the host response as well as the microbial taxa in tissues and their intratissue expression levels using metatranscriptomics in naturally infected tissues. It is well published that skin damage is required to allow D. nodosus infection to establish (2, 51). As expected, the host response in footrot is characterized by the differential expression of proteins with roles in wound healing and chronic wounds. As in the absence of D. nodosus, interdigital dermatitis resolves, the presence of D. nodosus may be essential to allow the establishment of the microbes associated with underrunning footrot, including *P. asaccharolytica*. In these later stages of the disease, the presence of those bacteria, such as M. fermentans, may contribute to a dampening of the immune response, which is unable to remove the invading bacterial pathogens, leading to chronic infection.

## MATERIALS AND METHODS

### Sample collection.

Sheep were assessed after slaughter for foot health. Any individual animals showing signs of footrot were selected for sample collection. Debris was removed from all feet, and the feet were cleaned using purified water. Sterile nylon flock swabs (E-swabs 480CE; Copan, USA) were taken from the interdigital space and stored in liquid Amies medium at 5 °C overnight. The foot was then washed with a chlorohexidine solution (National Veterinary Services, UK). Any hair was removed from the feet with scissors prior to the collection of an 8-mm biopsy specimen using a punch (National Veterinary Services, UK). Biopsy specimens were placed in RNAlater (Sigma-Aldrich, UK) and stored at 5 °C overnight before being frozen at −80 °C. This study was reviewed and approved by the University of Nottingham School of Veterinary Medicine and Science ethical review committee (ERN 1144 140506 [non-ASPA]).

### DNA extraction from swabs.

The interdigital space swabs were placed on a MixMate instrument (Thermo Fisher, UK) for 5 min at 800 rpm to thoroughly disperse the bacteria in the Amies solution from the swab. The liquid was transferred into a low-bind 1.5-ml tube and centrifuged at 12,000 rpm for 5 min. The supernatant was removed, and the pellets were resuspended in 200 μl of RNase-free molecular-biology-grade water (Thermo Fisher, UK) (52). DNA was isolated using the Qiagen Cador pathogen minikit, according to the manufacturer’s guidelines, and eventually eluted in 60 μl of elution buffer. The DNA samples were quantified using the Qubit 3.0 system and double-stranded DNA (dsDNA) high-sensitivity dye (Qiagen).

### RNA extraction.

Biopsy specimens were thawed on ice before being cut into approximately 30-mg sections. One section was added to a MACs M tube (Miltenyi Biotech, UK) containing 1 ml Qiazol (Qiagen, UK) and dissociated on a GentleMACs instrument (Miltenyi Biotech, UK) using the manufacturer’s RNA settings. The sample was centrifuged and incubated at room temperature (RT) for 5 min before transferring the lysate to a 1.5-ml centrifuge tube. Proteinase K (20 μl) was added to the sample before being incubated at 56 °C for an hour. Chloroform (200 μl) was added, and the mixture was shaken vigorously for 15 s. The sample was then incubated at RT for 2 min before being centrifuged at 12,000 × *g* for 15 min at 4 °C. The upper aqueous phase was transferred to a fresh 1.5-ml centrifuge tube before the addition of a 1× volume of 70 % ethanol. The sample (up to 700 μl) was added to an RNeasy minispin column (Qiagen, UK) and centrifuged at RT at 8,000 × *g* for 30 s. Any remaining sample was also passed through the column. All remaining steps were performed according to the manufacturer’s guidelines, with elution in 30 μl of RNase-free molecular-biology-grade water (Thermo Fisher, UK).

### Dual RNA sequencing.

The extracted RNA was quantified using the Agilent Bioanalyser RNA Nano 6000 kit. Healthy foot sample RNA with a RNA Integrity Number (RIN) score of ≥7 and footrot sample RNA with a DV200 of >85 were chosen for sequencing. The samples were treated using the RiboZero gold (epidemiology) ribosomal depletion kit (Illumina, USA) and prepared for sequencing using Illumina TruSeq library preparation (Illumina, USA). The samples were sequenced on a HiSeq 3000 instrument using 150-bp paired-end chemistry (Leeds Institute of Molecular Medicine Sequencing Facility) at 6 libraries per lane over 14 lanes, giving approximately 75 million reads per sample.

### Data analysis.

All analysis was carried out using default settings unless stated otherwise. Raw reads were analyzed for quality and adaptor removal using Skewer (53). An initial step for the RNA sequencing (RNA-seq) data consisted of aligning the reads with HISAT2 (54) against the sheep genome (Oar_v3.1, downloaded on 21 July 2017) (55) to separate the ovine and potential bacterial transcripts. The sheep reads were then parsed for transcript alignment using Kallisto (56) and the sheep genome (Oar_v3.1, downloaded on 21 July 2017) (55). Differential analysis was performed with Sleuth (57). The differentially expressed genes were imported into R (58) and clustered using BiClust and block-correlated coupled clustering (59). The reads that did not align to the sheep genome and the metagenomic reads were used as the input for taxonomic assignment, bacterial populations were determined with Kraken (60), false-positive results were identified with KrakenUniq (61), and results were filtered with MAG_TaxaAssigner (https://github.com/shekas3/BinTaxaAssigner).

### Confirmation of selected bacterial species by PCR or qPCR.

Parallel tissue samples from the same foot as for RNA isolation were used. Tissue homogenization and DNA extractions were performed as described previously using a QIAamp Cador kit (Qiagen) (62). The DNA samples were quantified using the Qubit 3.0 instrument and dsDNA high-sensitivity dye (Qiagen). The bacterial load was quantified using real-time PCR based on the 16S rRNA gene for eubacteria (63) and D. nodosus (64) and F. necrophorum subsp. *necrophorum* primers targeted to the *gyrB* gene (52). M. fermentans sequences were amplified by species-specific PCR for 16S rRNA (65), and *P. asaccharolytica* sequences were amplified by PCR (66) followed by sequencing (Eurofins Genomics).

### Correlation testing.

Both tests were performed using the same data set. For each of the samples (biopsy, swab, and host), each of the bacterial species was labeled as either present or absent. A difference score was prescribed if a species was present in one of the swab or biopsy samples from a sheep but not both (i.e., the swab and biopsy samples gave different results for the presence of the species). If present in both or neither the biopsy nor swab samples, a *t* test statistic was then calculated by taking the sum of all differences across all sheep. To perform the randomization procedure, the labels of the original 52 samples (sheep and sample type) were randomly reassigned, and the test statistic was recalculated. This procedure was repeated 10,000 times, giving randomized test statistics.

### Host-pathogen interactions.

To identify putative host-pathogen interactions, the correlation script PHInder was used (https://github.com/addyblanch/PHInder). Briefly, samples with zero assignments were removed, and the minimum presence value was set to 1. A new data matrix was formed, and the hypergeometric distribution was calculated using phyper (https://www.rdocumentation.org/packages/stats/versions/3.6.2/topics/Hypergeometric) and a probability, producing a significance (*P*) value and an adjusted *P* value.

### Tissue staining, image capture, and analysis.

In order to determine collagen types and quantities within each tissue, samples were processed, sectioned, stained, and imaged microscopically, and thereafter, each photomicrograph was analyzed. The tissue biopsy specimens were fixed, dehydrated, and mounted in paraffin blocks, and 7-μm-thick serial sections were collected throughout the tissue and placed onto polysilinated microscope slides. The paraffin surrounding each tissue section was removed by melting it at 60 °C for 5 to 10 min, followed by immersion in xylene (twice for 5 min). Tissue sections were then rehydrated in 100, 90, and 70 % ethanol and then in distilled water for 5 min each. Picrosirius (PS) red stain was used on each tissue section to differentiate and quantify collagen types I and III and undifferentiated collagens using the picrosirius stain kit (Polysciences, Inc., PA, USA). The observer was blinded to the sample identification to avoid subconscious bias. Images from each stained tissue section were captured using a Leica CTR500 microscope (Leica Microsystems, Germany) with and without polarized light at a ×40 magnification. For each sample, three PS-stained sections approximately 400 μm apart were analyzed using five nonoverlapping photos per dermis section (systematic random sampling).

The resulting 315 photomicrographs from 13 healthy and 8 footrot samples were analyzed using Image-Pro Plus (Media Cybernetics, Inc., PA, USA) to quantify the area of collagen in each image, with separate measurements for type III (green), type I (yellow), and undifferentiated (red) collagen. The total collagen proportion was calculated as the sum of type III, type I, and undifferentiated collagen proportions.

### Statistical analysis.

The taxonomic count data were analyzed for statistically significant differences in R (58) using the edgeR wrapper (67) as part of the Phyloseq package (68). Diversity statistics were calculated using vegan (69), and differences were calculated using Mann-Whitney U tests in Prism 8.01 (GraphPad Software, Inc., USA).

Statistical analyses of histology images were performed in GraphPad Prism version 6 for Windows. The resulting data are presented as frequencies and percentages and were analyzed by the Student *t* test or Kruskal-Wallis test, depending on the data distribution. Analysis was taken as significant when the *P* value was ≤0.05.

## DATA AVAILABLITTY

All sequence data generated for this study are held in the NCBI SRA under the accession number PRJNA725378.
